# The Role of Inflammatory Markers NLR and PLR in Predicting Pelvic Pain in Endometriosis

**DOI:** 10.3390/jcm14010149

**Published:** 2024-12-30

**Authors:** Oana Maria Gorun, Adrian Ratiu, Cosmin Citu, Simona Cerbu, Florin Gorun, Zoran Laurentiu Popa, Doru Ciprian Crisan, Marius Forga, Ecaterina Daescu, Andrei Motoc

**Affiliations:** 1 Doctoral School, “Victor Babes” University of Medicine and Pharmacy Timisoara, Eftimie Murgu Square 2, 300041 Timisoara, Romania; oana-maria.gorun@umft.ro; 2 Department of Obstetrics and Gynecology, “Victor Babes” University of Medicine and Pharmacy Timisoara, Eftimie Murgu Square 2, 300041 Timisoara, Romania; citu.ioan@umft.ro (C.C.); popa.zoran@umft.ro (Z.L.P.); crisan.doru@umft.ro (D.C.C.); forga.marius@umft.ro (M.F.); 3 Department of Orthopedics, Traumatology, Urology and Medical Imaging, Discipline of Radiology and Medical Imaging, “Victor Babes” University of Medicine and Pharmacy Timisoara, Eftimie Murgu Square 2, 300041 Timisoara, Romania; cerbu.simona@umft.ro; 4 Department of Obstetrics and Gynecology, Municipal Emergency Clinical Hospital Timisoara, 300172 Timisoara, Romania; gorun.florin@umft.ro; 5 Department of Anatomy and Embryology, “Victor Babes” University of Medicine and Pharmacy Timisoara, Eftimie Murgu Square 2, 300041 Timisoara, Romania; daescu.ecaterina@umft.ro (E.D.); amotoc@umft.ro (A.M.)

**Keywords:** endometriosis, inflammatory markers, pelvic pain, NLR, PLR

## Abstract

**Background/Objectives**: Chronic inflammation plays a critical role in pelvic pain among endometriosis patients. This study examines the association between inflammatory markers—specifically the neutrophil-to-lymphocyte ratio (NLR) and platelet-to-lymphocyte ratio (PLR)—and pelvic pain in endometriosis. **Methods**: We conducted a retrospective analysis of endometriosis patients, assessing NLR and PLR levels in those with and without pelvic pain. Diagnostic utility was evaluated using ROC curves, and logistic regression determined associations between these markers, pain presence, and endometriosis severity. **Results**: Patients with pelvic pain had significantly higher median levels of both NLR and PLR (*p* < 0.05). NLR demonstrated moderate diagnostic accuracy with an AUC of 0.63, sensitivity of 59%, and specificity of 71% at a cut-off of 1.85. PLR, with a cut-off of 139.77, showed an AUC of 0.60, with a specificity of 82% and sensitivity of 40%, indicating better utility for excluding pain. Logistic regression analysis revealed that NLR > 1.85 was significantly associated with pelvic pain (OR = 3.06, 95% CI: 1.45–6.49, *p* = 0.003), as was PLR > 139.77 (OR = 2.84, 95% CI: 1.18–6.82, *p* = 0.02). Advanced rASRM stages (III and IV) also correlated with elevated NLR and PLR values. **Conclusions**: Elevated NLR and PLR are associated with pelvic pain and advanced stages of endometriosis, suggesting these ratios are potential markers for assessing inflammation and disease severity. Further studies should explore combining NLR and PLR with other biomarkers to improve diagnostic accuracy in endometriosis.

## 1. Introduction

Endometriosis is an increasingly common gynecological condition among women, affecting up to 10% of women of reproductive age. This estrogen-dependent pathology is defined by the presence of endometrial-like tissue outside the uterine cavity, causing an inflammatory response with a variety of consequences that can affect quality of life, such as pelvic pain, dysmenorrhea, dyspareunia, and infertility [1,2,3].

Endometriosis is most commonly found in the pelvic area: on the ovaries, uterus, fallopian tubes, uterosacral ligaments, large ligaments, round ligaments, cul-de-sac, rectosigmoid colon, bladder, ureters, and rectovaginal septum. As extra-pelvic sites, thoracic-diaphragmatic endometriosis or sciatic nerve endometriosis may rarely occur [4].

The gold standard for diagnosing endometriosis remains laparoscopy, an invasive method that is only indicated in women with relevant symptoms. Thus, without the availability of non-invasive diagnostic tests, the average time from onset of symptoms to definitive diagnosis of endometriosis is 7–8 years [4,5].

The most debilitating symptom of endometriosis is pain, which has proven to be very difficult to manage, manifesting at both somatic and visceral levels: dysmenorrhea, pelvic pain, dysuria, dyspareunia, radiating low back pain, dyskinesia. Extensive research has found that inflammatory response is key to the pathogenesis of endometriosis. Therefore, the association of endometrial lesions with inflammatory components leads to pain [6,7,8,9].

The chronic inflammatory nature of endometriosis plays a central role in the pathogenesis and symptomatology of this condition. Chronic inflammation is a common feature in many systemic diseases, including cardiovascular diseases, autoimmune disorders, and metabolic syndromes, resulting from persistent activation of the immune system. In endometriosis, elevated levels of proinflammatory cytokines, chemokines, and reactive oxygen species are observed in the peritoneal fluid and in the lesion microenvironment [8,10].

Furthermore, chronic inflammation contributes to angiogenesis and fibrosis, which are necessary processes in the establishment and progression of ectopic endometrial lesions. Interactions between immune cells and endometrial lesions amplify the inflammatory cycle, hence emphasizing pain and other symptoms [11].

Inflammatory biomarkers such as neutrophil-to-lymphocyte ratio (NLR) and platelet-to-lymphocyte ratio (PLR) have emerged as promising tools for assessing systemic inflammation in chronic diseases, including endometriosis. These accessible markers provide information about the inflammatory status of patients and may correlate with disease severity and symptomatology. NLR and PLR have been studied in various chronic inflammatory conditions, such as rheumatoid arthritis and cardiovascular disease, where they are associated with disease activity and prognosis [12]. These markers are inexpensive, accessible, and widely used in clinical practice. In endometriosis, they may provide insight into the inflammatory burden and disease severity, particularly in relation to pelvic pain, a debilitating symptom experienced by many patients [9,13,14,15].

This study aimed to compare NLR and PLR levels in endometriosis patients to better understand the role of inflammation in relation to pelvic pain in this condition.

## 2. Materials and Methods

### 2.1. Study Design and Setting

This retrospective, cross-sectional study was conducted at a single center and included patients diagnosed with and hospitalized for endometriosis at the Timisoara Municipal Emergency Hospital between 1 January 2017 and 31 December 2022. Data were retrospectively collected from the hospital’s electronic medical records system. The dataset included demographic information (age, residence), clinical symptoms (pelvic pain), laboratory test results (complete blood count and inflammatory markers), surgical results, and histopathologic confirmation of endometriosis. Each record was independently reviewed by two investigators to ensure accuracy and completeness.

The study was approved by the Ethics Committee of the University of Medicine and Pharmacy, “Victor Babes” Timisoara (No. 22726, 17 November 2021), and it was conducted in accordance with the STROBE guidelines [16].

### 2.2. Participants

Participants that were included fulfilled the following requirements: (1) confirmed histopathologic diagnosis of endometriosis following surgery; (2) hospital admission at the Timisoara Municipal Emergency Hospital between 1 January 2017 and 31 December 2022; (3) documented complete blood count at the time of admission; and (4) women at reproductive age, between 18 and 50 years.

**Figure 1:**
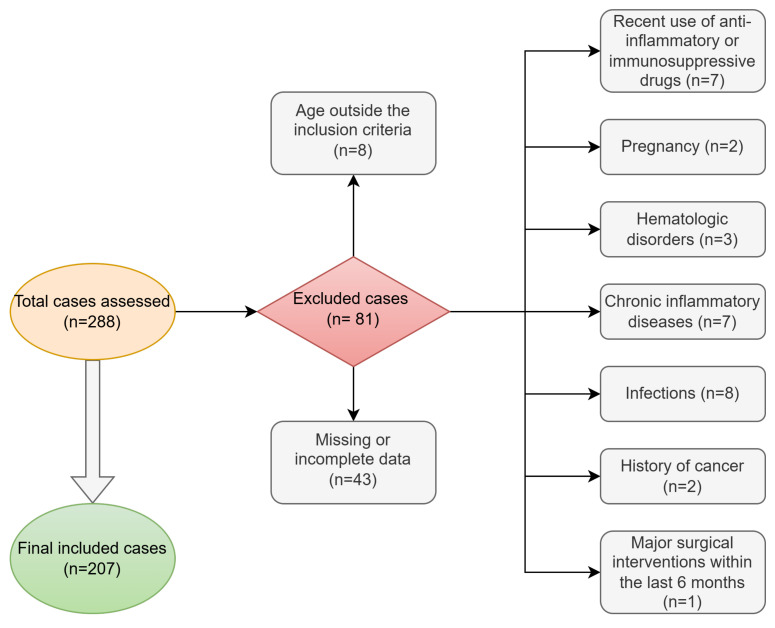
Flowchart of case selection for the study.

Excluded cases were based on the following criteria: (1) presence of chronic inflammatory diseases (rheumatoid arthritis, chronic obstructive pulmonary disease, autoimmune thyroiditis); (2) active infections at the time of hospitalization (urinary tract infection, upper respiratory tract infection); (3) hematologic disorders affecting blood cell counts (anemia, thrombocytopenia); (4) recent use of anti-inflammatory or immunosuppressive drugs; (5) history of cancer; (6) incomplete medical records, including missing blood tests, histopathologic result or clinical data; (7) pregnancy; and (8) major surgical interventions within the last 6 months (Figure 1).

### 2.3. Variables, Data Sources, and Measurement

Data were extracted from patients’ electronic medical records and organized using a standardized data collection form. Demographic, clinical, and laboratory data collected included age, history, presence or absence of pelvic pain, endometriosis localization, rASRM (revised American Society for Reproductive Medicine) score, endometriosis stage according to rASRM classification, and complete blood count (CBC) at the time of hospital admission. Inflammatory biomarkers were calculated from the initial CBC using the following formulas: NLR = absolute neutrophil count (ANC)/absolute lymphocyte count (ALC) and PLR = absolute platelet count (APC)/ALC. Endometriosis staging and classification according to the revised American Society for Reproductive Medicine (rASRM) system were as follows: Stage I (minimal, 1–5 points), Stage II (mild, 6–15 points), Stage III (moderate, 16–40 points), and Stage IV (severe, >40 points).

### 2.4. Statistical Analysis

Data were analyzed using SPSS 20.0 software (SPSS Inc., Chicago, IL, USA) and Python version 3.11 (https://www.python.org, accessed on 15 September 2024). Continuous variables were assessed for normality using the Shapiro–Wilk test. Descriptive statistics for continuous variables were expressed as medians and interquartile ranges (IQR) due to non-normal distribution. Categorical variables were presented as frequencies and percentages.

For comparison between groups (“with pelvic pain” and “no pelvic pain”), the Mann–Whitney U test was applied to assess differences in continuous variables, such as NLR, PLR, and ASRM score.

Spearman’s correlation was used to assess the association between inflammatory biomarkers (NLR, PLR) and clinical variables, including pelvic pain and ASRM stage. Logistic regression analysis was performed to evaluate the predictive value of NLR, PLR, ASRM score, and age for the presence of pelvic pain. Model performance was assessed using the area under the ROC curve (AUC) to evaluate classification accuracy.

A *p*-value of <0.05 was considered statistically significant for all analyses.

## 3. Results

### 3.1. Participants Characteristics

A total of 207 patients diagnosed with endometriosis were enrolled in the study and evaluated based on clinical, demographic, and laboratory data. The median (IQR) age of the patients was 33 (10) years, with an age range between 18 and 50 years. All patients had histopathologically confirmed endometriosis following surgical intervention. Among these patients, 169 (81.6%) reported experiencing pelvic pain, while 38 (18.4%) did not report pelvic pain. Patients with pelvic pain had significantly higher NLR and PLR compared to those without pain. The median (IQR) NLR for patients with pelvic pain was 2.01 (1.35) versus 1.50 (0.98) for patients without pain (*p* = 0.011). Similarly, the PLR was higher in patients with pain (median 123.50) compared to those without (median 112.28; *p* = 0.048). Additionally, the median rASRM score, reflecting endometriosis severity, was significantly higher in the pelvic pain group (16) compared to the no pain group (5), with a highly significant *p*-value of 0.000003. However, no significant difference was observed in age or platelet count between the two groups.

The laboratory findings, including median values and interquartile ranges, are summarized in Table 1. These results suggest a potential link between higher inflammatory marker ratios (NLR, PLR) and the presence of pelvic pain in endometriosis, while ASRM scores indicate a possible association between pain and disease severity.

### 3.2. Comparison of Inflammatory Markers by Pelvic Pain Status in Endometriosis Patients

Figure 1 shows that patients with pelvic pain have higher median levels of both NLR and PLR than those without pain, suggesting an increased inflammatory response. The significant differences in NLR (*p* = 0.011) and PLR (*p* = 0.048) highlight these markers as potential indicators of pain-related inflammation in endometriosis. Elevated NLR and PLR may reflect underlying pain-related inflammatory activity in endometriosis, thus providing insight into symptom severity and aiding in pain assessment and management.

### 3.3. ROC Analysis of NLR and PLR Cut-Offs in Endometriosis-Related Pain

**Figure 2:**
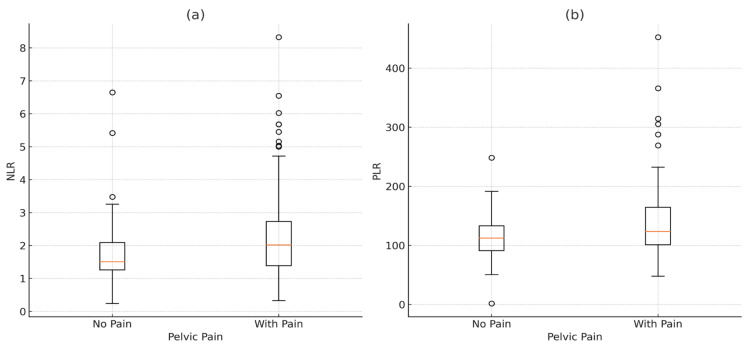
Distribution of inflammatory markers (NLR and PLR) by pelvic pain status in endometriosis patients: (**a**) NLR (neutrophil-to-lymphocyte ratio) distribution; (**b**) PLR (platelet-to-lymphocyte ratio) distribution.

Receiver operating characteristic (ROC) curves for NLR and PLR were generated to assess whether the baseline values of each of these biomarkers are predictive of the presence of pain in patients with endometriosis (Figure 2).

The areas under the curve (AUC) of NLR and PLR were 0.63 and 0.60, respectively. The optimal cutoff values obtained from Youden’s index are shown in Table 2. Both markers demonstrated high positive predictive values (>90%), indicating good capacity to confirm pelvic pain, but low negative predictive values (<28%), which limit their utility for excluding pain.

### 3.4. Association of NLR, PLR, rASRM Stage with Pelvic Pain

A univariate regression analysis was conducted to determine the relationship between inflammatory biomarkers and pelvic pain. Both NLR and PLR (above or below cut-off values) were significant predictors of pelvic pain in the analysis (Table 3).

In addition, a multivariate logistic regression was performed to assess the discriminatory ability of NLR (above or below cut-off values) as a prognostic factor for pelvic pain, adjusted for rASRM stages. The results showed an odds ratio (OR) of 2.32 for NLR above 1.85, 1.16 for rASRM Stage II, 11.92 for Stage III, and 9.31 for Stage IV (Table 4).

Similarly, a multivariate logistic regression was performed to assess the discriminatory ability of PLR (above or below cut-off values) as a prognostic factor for pelvic pain, adjusted for rASRM stages. The results showed an OR of 2.45 for PLR above 139.77, 1.11 for rASRM Stage II, 12.06 for Stage III, and 11.12 for Stage IV (Table 5).

### 3.5. Inflammatory Markers Relationship with Endometriosis Site and Severity

**Figure 3:**
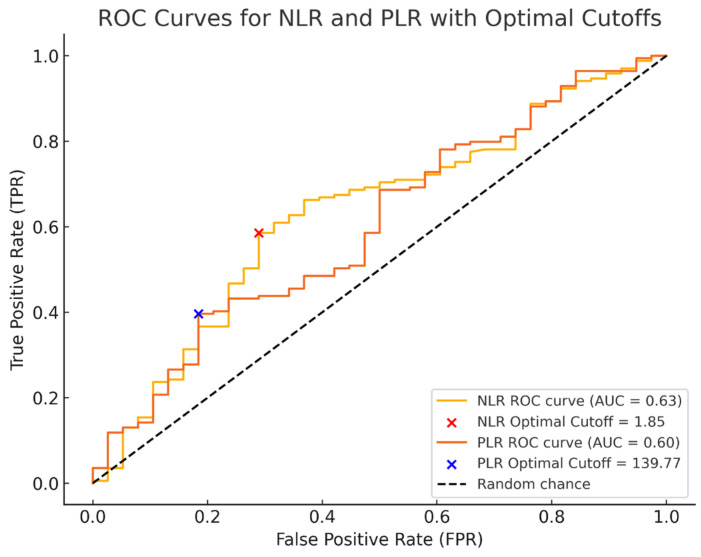
Receiver operating characteristic (ROC) curves for NLR and PLR with optimal cutoff points in predicting pelvic pain.

The analysis of NLR and PLR distributions across different endometriosis sites did not reveal statistically significant differences, as indicated by the Kruskal–Wallis test (*p* > 0.05). This suggests that both NLR and PLR values are consistent across sites, showing no specific pattern related to the location of endometriosis (Figure 3).

**Figure 4:**
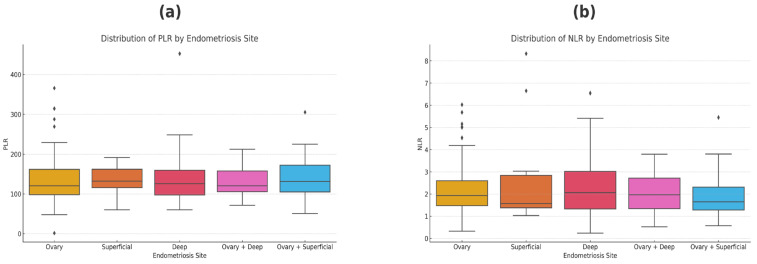
Distribution patterns of NLR and PLR in different endometriosis sites: (**a**) distribution of PLR and (**b**) distribution of NLR.

The median values of both inflammatory markers are similar between endometriosis localizations (ovarian, superficial, deep, etc.), and the variability is also uniform. The presence of outliers suggests a more pronounced inflammatory response in some patients, but these do not affect the overall distribution (Figure 4).

**Figure 5:**
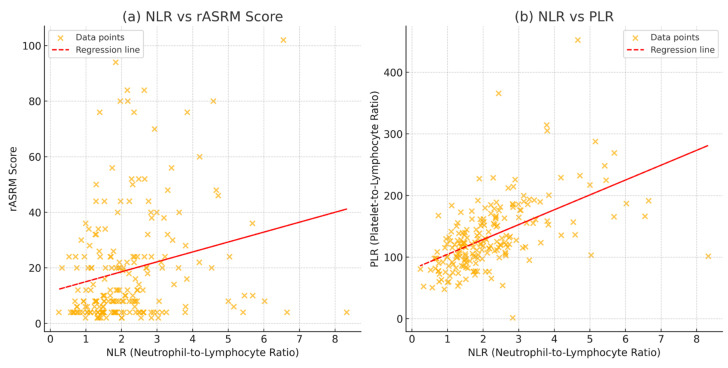
Correlation between inflammatory markers (NLR and PLR) and endometriosis severity (ASRM score): (**a**) correlation between NLR and ASRM score and (**b**) correlation between NLR and PLR.

Spearman’s rank correlation was computed to assess the relationship between NLR, PLR, and ASRM scores (Figure 5). There was a moderate to strong positive between NLR and PLR with a correlation coefficient of 0.608 and a *p*-value = 0.000. Between the NLR and the ASRM score, a weak positive correlation is observed, with a correlation coefficient of 0.245 and *p*-value = 0.000.

## 4. Discussion

Chronic inflammatory response appears to be an important cause of pelvic pain in patients with endometriosis. Therefore, in this study, we investigated the association between inflammatory markers, in particular, the neutrophil-to-lymphocyte ratio (NLR) and the platelet-to-lymphocyte ratio (PLR), with the presence of pelvic pain in patients with endometriosis.

Our results showed that patients with pelvic pain have higher median levels of both NLR and PLR compared to those without pain. The statistically significant differences observed for NLR and PLR emphasize the potential of these markers as indicators of pain-related inflammation in endometriosis. Similarly, Jing et al. reported that NLR and PLR values are higher in patients with symptomatic endometriosis [2]. In contrast, Yavuzcan et al. found no significant differences in NLR and PLR between patients with endometriosis and two comparison groups without endometriosis, which may be attributed to the study’s small sample size [17].

We acknowledge that NLR and PLR are non-specific markers influenced by factors such as age, hydration status, and stress, which can challenge the establishment of universal cut-off points for disease severity. However, our findings demonstrate their potential utility in identifying inflammatory profiles in patients with endometriosis, supporting their role as complementary tools rather than standalone diagnostic measures. Furthermore, in the multivariate analysis, we included age alongside other variables, demonstrating that NLR > 1.85 and PLR > 139.77 were significantly associated with pelvic pain, highlighting their relevance in disease severity.

Inflammatory markers such as NLR and PLR have also been investigated in other inflammatory diseases beyond endometriosis. Scalise et al. demonstrated the role of inflammation biomarkers, including NLR and PLR, in predicting outcomes in carotid artery stenosis procedures. Their study highlighted that elevated NLR levels were associated with increased perioperative risk and complications, supporting the systemic nature of inflammation in chronic conditions [18]. Similarly, Costa et al. explored how omics science integrates inflammatory biomarkers for diagnosis and outcome prediction in carotid stenosis. Their findings emphasize that combining inflammatory markers with omics approaches enhances the accuracy of diagnosis and prognosis in inflammatory-driven diseases [19].

When evaluating the diagnostic utility of NLR and PLR for identifying pelvic pain in patients with endometriosis, NLR demonstrated moderate diagnostic accuracy with an AUC of 0.63, a sensitivity of 59%, and a specificity of 71% (cut-off 1.85), while PLR with a cut-off of 139.77, showed an AUC of 0.60, with a specificity of 82% and a sensitivity of 40%, indicating that it is better at excluding pain than detecting it, thus limiting its independent diagnostic value. Moreover, studies have concluded that NLR and PLR combined with CA-125 were significantly higher compared to using each marker independently [20,21].

Azizoglu et al. investigated the utility of inflammatory ratios in predicting surgical interventions in pediatric adhesive small bowel obstruction, concluding that ratios such as bilirubin-to-lymphocyte could effectively reflect systemic inflammation [22]. Similarly, Aydoğdu et al. developed a diagnostic scoring system for pediatric appendicitis using hematological parameters, showing that inflammation markers provide significant diagnostic insight [23]. These findings further underline the utility of systemic inflammatory markers like NLR and PLR in identifying inflammation-related pathologies across different conditions.

The logistic regression analysis revealed that both NLR and PLR are significantly associated with the presence of pelvic pain in patients with endometriosis. The results indicate that an NLR value greater than 1.85 is significantly associated with a higher likelihood of experiencing pelvic pain, with an OR of 3.06 (*p* = 0.003; 95% CI: 1.45–6.49). Moreover, a PLR value greater than 139.77 was associated with a significantly higher likelihood of pelvic pain, with an OR of 2.84 (*p* = 0.02; 95% CI:1.18–6.82). In line with our results, Dominoni et al. highlighted the association between NLR and chronic pelvic pain (OR = 3.9; CI: 1.2–12.3; *p*-value = 0.02), suggesting that elevated NLR may reflect the inflammatory response contributing to pain severity [24]. Additionally, other studies showed an increase in PLR in the endometriosis group, further supporting its role as an inflammatory marker in this condition [9,25,26].

Furthermore, when considering rASRM stages, advanced stages (III and IV) were significantly associated with an increased likelihood of pain for both inflammatory markers; thus, stage III had a strong association with pelvic pain, with an OR of approximately 12 (NLR: 11.92, PLR: 12.06), likewise stage IV showed high odds ratios in both analyses (NLR OR: 9.31; PLR OR: 11.12). However, the confidence intervals for stage IV are wide in both analyses, especially for PLR (1.39–88.73), reflecting higher variability, possibly due to a smaller sample size at this stage. Supporting our findings, studies conducted by Yang et al. and Cho et al. demonstrated that NLR and PLR have higher values in moderate to severe endometriosis, highlighting that these inflammatory markers might reflect the severity in the advanced stages of this condition [27,28]. In addition, Cepeda-Silva et al. explored colorectal endometriosis, proposing a complementary classification based on surgical staging. Their findings further reinforce the importance of inflammation in determining disease severity and surgical outcomes [29]

Our study further demonstrated that elevated NLR is associated with higher rASRM scores, indicating higher disease severity, and with increased PLR, suggesting increased systemic inflammation. Thus, the positive correlation between NLR and ASRM score reinforces the potential of NLR as a marker for assessing endometriosis severity, and the correlation between NLR and PLR suggests that these markers may together reflect the inflammatory nature of endometriosis [20]. On the other hand, NLR and PLR presented a relatively similar distribution among different endometriosis localizations and did not show statistically significant differences. Tokmak et al. also did not find statistical differences in endometriosis sites, indicating that these markers are more reflective of general inflammation rather than site-specific disease characteristics [20].

The strengths of this study include addressing the clinically relevant problem by evaluating the potential of NLR and PLR as accessible biomarkers for detecting pain-related inflammation in endometriosis. Also, the identification of reliable and cost-effective markers is valuable for improving the management of endometriosis. In addition, the study utilizes robust statistical methods, including logistic regression and ROC curve analysis, to further evaluate the diagnostic utility of NLR and PLR.

However, some limitations should be considered. The retrospective, single-center design of the study may limit the applicability of the findings. In addition, the cross-sectional nature of the data limits our ability to make causal conclusions regarding the relationship between elevated NLR/PLR levels and pelvic pain in endometriosis. Moreover, NLR and PLR are non-specific markers influenced by factors such as age, hydration status, and stress, which can challenge the establishment of universal cut-off points for disease severity. Another limitation is the relatively smaller sample size for patients with advanced stages of endometriosis, which may contribute to variability in the confidence intervals for stage IV results. Finally, while this study focused on NLR and PLR individually, it did not assess their combined potential with other biomarkers, such as CA-125, which could potentially enhance diagnostic accuracy. Future studies should consider these limitations when exploring the diagnostic potential of these markers.

## 5. Conclusions

In summary, our findings highlight the utility of NLR and PLR as systemic markers of inflammation in endometriosis, particularly in predicting pain and disease severity. Both markers provide important information about the inflammatory nature of endometriosis, but NLR showed a slightly higher correlation with disease severity than PLR. To increase diagnostic accuracy, future research should examine these markers in combination with more specialized inflammatory biomarkers.

## Figures and Tables

**Table 1:** Baseline characteristics of 207 patients with endometriosis.

Variables	Total Median (IQR)	Pelvic Pain Median (IQR)	No Pelvic Pain Median (IQR)	*p*-Value
Demographics				
Age	33.00 (10)	33.00 (10)	32.50 (12)	0.844
Laboratory findings				
Neutrophils (10^3^/µL)	4.15 (2.40)	4.23 (2.47)	3.60 (1.87)	0.099
Lymphocytes (10^3^/µL)	2.13 (0.86)	2.10 (0.81)	2.40 (1.185)	0.029
Platelets (10^3^/µL) Inflammatory markers	273.00 (93.00)	274.00 (98.00)	267.50 (72.75)	0.697
NLR PLR	1.92 (1.31) 122.08 (63.49)	2.01 (1.35) 123.50 (63.73)	1.50 (0.98) 112.28 (47.06)	0.011 0.048
rASRM score	10.00 (20)	16 (24)	5.00 (5)	<0.001

NLR = neutrophil-to-lymphocyte ratio; PLR = platelets-to-lymphocyte ratio; rASRM = revised American Society for Reproductive Medicine.

**Table 2:** AUC-ROC of inflammatory biomarkers and optimal cut-off.

Variable	Cut-Off	AUC	Youden	Sensitivity	Specificity	PPV	NPV
NLR	1.85	0.63	0.30	59%	71%	90.0%	27.83%
PLR	139.77	0.60	0.21	40%	82%	90.54%	23.31%

AUC = area under the curve; PPV = positive predictive value; NPV = negative predictive value.

**Table 3:** Univariate binominal logistic regression analysis.

Variables	B	Std. Error	Odds Ratio	*p* Value	Confidence Interval 95%	
					Lower	Upper
NLR > 1.85	1.12	0.38	3.06	0.003	1.45	6.49
PLR > 139.77	1.04	0.44	2.84	0.02	1.18	6.82

B = unstandardized beta coefficient; NLR = neutrophil-to-lymphocyte ratio.

**Table 4:** Multivariate binominal logistic regression analysis for NLR.

Variables	B	Std. Error	Odds Ratio	*p* Value	Confidence Interval 95%	
					Lower	Upper
NLR > 1.85	0.84	0.40	2.32	0.037	1.05	5.13
rASRM stage I	Ref	Ref	Ref	Ref	Ref	Ref
rASRM stage II	0.15	0.41	1.16	0.71	0.52	2.60
rASRM stage III	2.48	0.78	11.92	0.001	2.61	54.49
rASRM stage IV	2.23	1.07	9.31	0.037	1.15	75.40

B = unstandardized beta; NLR = neutrophil-to-lymphocyte ratio; rASRM = revised American Society for Reproductive Medicine; CI = confidence interval.

**Table 5:** Multivariate binominal logistic regression analysis for PLR.

Variables	B	Std. Error	Odds Ratio	*p* Value	Confidence Interval 95%	
					Lower	Upper
PLR > 139.77	0.89	0.47	2.45	0.037	0.97	6.15
rASRM stage I	Ref	Ref	Ref	Ref	Ref	Ref
rASRM stage II	0.11	0.41	1.11	0.71	0.49	2.50
rASRM stage III	2.49	0.77	12.06	0.001	2.64	55.03
rASRM stage IV	2.41	1.06	11.12	0.037	1.39	88.73

B = unstandardized beta; PLR = platelets-to-lymphocyte ratio; rASRM = revised American Society for Reproductive Medicine; CI = confidence interval.

## Data Availability

The data sets used and/or analyzed during the present study are available from the correspondence author on reasonable request.
